# Microbial Musings – Winter 2022

**DOI:** 10.1099/mic.0.001310

**Published:** 2022-12-31

**Authors:** Gavin H. Thomas

**Affiliations:** ^1^​ Department of Biology, University of York, York, UK

## Full-Text

As we close another year with microbiology still on our minds through continued circulation of SARS-COV-2 variants, a bacterial pathogen has now come into the public’s attention in the UK with a number of deaths of children linked to *Streptococcus pyogenes* infections (Strep A). This bacterium is the cause of scarlet fever, a disease that has massively reduced in occurrence in the twentieth century, but is carried as a commensal by up to 15 % of children [1]. As well as other diseases milder than scarlet fever, infection by the bacterium can also lead to rarer cases of invasive disease, which was the cause of the tragic child deaths in the UK. The bacterium generally responds well to antibiotic treatment in mild cases, which combined with a large decrease in the occurrence of scarlet fever has removed this pathogen from most people’s attention, but clinicians and microbiologists recognize that it is still able to cause explosive outbreaks and remains circulating in the general population [1].

A cousin of *S. pyogenes* is the feature of a new Microbe Profile and is another important human pathogen, that similarly was a major cause of mortality under the advent of antibiotics. This bacterium, *Streptococcus pneumoniae *, inhabits the upper respiratory tract of humans, often asymptomatically, but can then spread to other body sites and cause pneumonia, meningitis or otitis media (inner ear infection). The biology of this bacterium is described by Hasan Yesilkaya, Marco Oggiani (@mr_oggioni) and Peter Andrew from the University of Leicester, UK, who very clearly summarize its key features [2]. It has a smallish genome of around 2 Mb, which encodes genes for a mainly sugar-based fermentative metabolism. The cells are usually seen as diplococci in spherical or lancet shapes (they look like two arrow heads pointing away from each other) and are non-motile. A key feature is their capsule layer, for which over 100 different biochemical forms are known. Capsular (smooth) and lab-isolated acapsular (rough) derivatives were indeed the basis of Griffith’s landmark work on transformation in the 1920s [3] after the bug had been first isolated by Pasteur back in 1881. Its genetic plasticity is another feature, which is mainly recombination based, allowing it to rapidly change capsule loci for example.

The ability of streptococci to evolve rapidly also is aided by their high level of genetic competency and this links to another paper in this quarter on the mechanisms of this process in the related species *Streptoccocus sinensis*, another of the Mitis group of streptococci [4], but one which is able to infect heart valves in humans (endocarditis). The process of genetic competence is well understood in *S. pneumoniae *, where a competence-stimulating peptide (CSP) is the signal in a quorum-sensing-based mechanism [5]. In this study from Alec Brennan, Anthony Harrington and colleagues in the group of Ytfah Tal-Gan at the University of Nevada, Reno (@unevadareno), USA, the authors wished to understand how similar this process is in *S. sinensis *. Typically in these systems a small propeptide, ComC, is secreted by the ComAB ABC transporter and accumulates in the immediate environs of the bacteria to be sensed by a receptor ComD and which subsequently activates a transcription factor ComE, that controls expression of other genes in a cascade, which leads to full competency within about 15 min. Here they first identify the processed ComC peptide, the CSP., by examining predictions from the genomes of *S. sinensis* and assessing peptides found in cell-free supernatant using mass spectrometry. They find a 16 amino peptide that shares similarity with other Mitis group streptococci, although there is significant sequence diversity amongst the different species. This CSP is released from early to mid-log phase as judged by the activation of expression of the *comX* gene that is switched on by the quorum-sensing pathway being activated. They show that synthetic CSP from their strain-induced competence, but this was not observed using a distinct CSP from a related strain, demonstrating high specificity of this sensing mechanism. To see which other genes are switched on they isolated RNA from matched cultures with added synthetic CSP or a DMSO control and used RNAseq to determine the differentially expressed genes. They find many of the genes they would have predicted to be involved in competence development, as expected, but also other genes involved in biofilm production and virulence. However, they directly assess biofilm formation in response to different concentrations of CSP and see no direct impact on this phenotype, suggesting there is additional regulatory control of biofilm formation, and they propose the differential regulation of some of the genes involved in this pathway by CSP might have become relevant during growth in the natural polymicrobial oral cavity.

Sticking with the streptococci for a final paper on this genus of bacteria, we switch to the work of *Microbiology* Editor Andy Edwards (@bugsinblood) and his team at Imperial College, London [6]. Authors Alicia Tickle and Elizabeth Ledger continue some research examining the effect of human serum on antibiotic resistance of important pathogens, following their earlier work on *Staphylococcus aureus* [7]. They examine the response of streptococci to the membrane targeting antibiotic daptomycin [8] and find the pre-exposure of the viridans group streptococci (VGS), namely, *Streptococcus gordonnii* and *Streptococcus mutans *, as well as related enterococci, to human serum, produces a high level of tolerance [9]. They knew from their work in *S. aureus* that serum exposure leads to peptidoglycan accumulation and this was also observed in the bacteria studied in this paper. The lipid cardolipin was also implicated in the serum resistance mechanism, with a reduction in the effect when both cardiolipin synthases were removed, which likely has a pleitrophic effect on many membrane functions and this reduction was found to be independent from that of peptidoglycan overproduction. Together these data suggest mechanisms for the lack of efficacy of daptomycin in the body, mediated by serum-induced responses in multiple bacteria. However, their data also suggest routes to enhance daptomycin killing *in vivo* by, for example, the use of fosfomycin to inhibit the overproduction of peptidoglycan.

One phenotype of bacterial cells that has come under much study and scrutiny is the phenomenon of persistence, namely the process where under some strong and sustained stress, such as antibiotic treatment, a subpopulation of cells survive and can grow again when the stress is removed. Xiaoyi Shi and Ashraf Zarken from the University of Cambridge, UK, present a detailed and critical analysis of the literature covering the different known triggers of persister formation, ranging from toxin-antitoxin modules, indole signalling and quorum sensing, which together suggest that multiple routes are likely to lead to persister formation [10]. In this wide-ranging review they also consider the nature of persister formation as a stochastic or deterministic process and the potential involvement of epigenetic mechanisms. Finally, they assemble a table of pathogens and clinically relevant antibiotics and argue that these are the combinations that should be used to learn about persisters in more clinical settings. Reflecting on a key methodological step in the study of persisters, namely the isolation of surviving sub-populations, a related Methods papers was also published in this quarter outlining a flow-cytometry protocol to identify *Pseudomonas aeruginosa* persister cells [11]. The protocol, from Shannon Grandy, Renee Raudonis and Zhenyu Cheng at Dalhousie University, Canada, presents their analysis of the best dyes to use with different strains of *P. aeruginosa* for the identification and isolation of antibiotic-induced persister cells. The staining and sorting can distinguish persisters from dead cells, however, they do note that even with improved recovery of persisters, they were unable to routinely regrow them on agar or in broth culture when isolated after long (24 h) treatment times with antibiotics, and while the media does make a difference in recovery rates, they propose that perhaps for cells in the persister states for this longer duration, addition cues beyond simple nutritional ones might be required.

Next up is a nice review from Michelle Kammel [12], which follows on from two papers published in the journal of her work in Gary Sawer’s group in Halle, Germany, on the mechanisms by which enterobacteria control the metabolic fate of formate [13, 14]. Briefly, this small organic acid is a major fermentation product of the enterobacterial mixed-acid fermentation and once made in the cytoplasm has two possible fates depending on other physiological factors in the cells. One of these uses formate in the cytoplasm and the other formate in the periplasm and so the FocA channel has a key role in controlling this partitioning of the substrate, and Kammel describes the latest research in understanding the function and regulation of this pentameric channel protein with some beautiful illustrations. Continuing the theme of the controlled movement of small molecules across membranes, transport, I want to unashamedly mention a paper from Adam Hughes and colleagues from the group of Anthony Wilkinson, from the York Structural Biology Laboratory (@YSBL_York), York, UK, as its back story is a nice example of a serendipitous observation leading into some nice insight into the evolution of ligand-binding specificity in proteins. I have been working with Tony for over a decade, always slightly awed by knowing that his group was the first to figure out how a general peptide transporter, in this case the Opp oligopeptide transporter from *Salmonella enterica* subsp. Typhimurium, was able to recognize almost any peptide between two to five amino acids by using only interactions between the main chain of the peptide ligand and the OppA protein and accommodating the variable amino acid side chains in water-filled pockets [15, 16]. Later we worked together to understand how a protein related to the OppA protein, known as a substrate-binding protein (SBP), was able to bind a specific peptide, in this case the murein tripeptide (Mtp) that is released in the periplasm of *Escherichia coli* during normal growth and then recycled [17]. This protein, MppA, which originated through duplication of *oppA* and subsequent modification, now recognizes specifically the Mtp and murein tetrapeptide [18] over any other peptide by using specific protein-ligand contacts that are selected against in the ‘generalist’ OppA protein. Jump forward a few years and Adam Hughes in Tony’s lab included some peptide-binding SBPs to study in his PhD project on the structural biology of proteins involved in sporulation in *Bacillus subtilis *. DppE is a known peptide-binding SBP that was named after its apparent ability to bind and transport dipeptides, however, Adam discovered that when he crystalized it there was density in the binding site that was unambiguously Mtp! He showed the protein really did bind Mtp and we did some genome context analysis and realised the transporter genes sit with other genes we had previously implicated in Mtp recycling [19]. Hence by comparing by MppA and DppE to the ancestral OppA we could see that both proteins had evolved to introduce the required specific contacts to become Mtp specific, but had changed different residues in the protein to create the same binding site through different evolutionary trajectories – a clear example of convergent evolution of a biochemical function.

We close with a slightly more unusual, but likely highly referenced, publication from Abigail Bartlett and colleagues including Daniel Padfield (@padpadpadpad) in the group of Michiel Vos (@Michiel_Vos_010) at the University of Exeter, UK, who have completed a major task in assembling in a single publication a list of all bacteria that have been reported to infect humans [20]. Although most of us can probably easily think of a few dozen pathogens, and medically minded microbiologists perhaps a few hundred, but they find over 1500 bacterial species implicated in human disease. About three quarters of these are ‘established’ pathogens, while the remainder they class as ‘putative’ as they have been found in fewer than three studies. What is fascinating is that these bacteria span 10 phyla and 24 classes of the bacterial world. They finish their mammoth task with a request – can somebody take on the task of converting this into a database and keeping this up to date for microbiologists? Sounds like a fantastic opportunity for some open science and collaborative and outreach opportunity that the Microbiology Society might support?

We will be back in 2023 for more microbiology news and commentary when the journal moves to full open access (OA) meaning that everybody can read both the Musings and all the papers that are being described for free! Exciting times.
